# Predicting Home Exercise Adherence after Ischemic Stroke: Development and Validation of a Web‐Based Nomogram

**DOI:** 10.1002/brb3.71482

**Published:** 2026-05-11

**Authors:** Wenbo Li, Qiujie Li

**Affiliations:** ^1^ Department of Clinical Nursing Education The Second Affiliated Hospital of Harbin Medical University Harbin China

**Keywords:** exercise therapy, nomograms, patient compliance, stroke rehabilitation

## Abstract

**Background:**

Poor adherence to home‐based functional exercises can substantially hinder recovery after ischemic stroke. This study aimed to develop and validate a web‐based predictive nomogram to identify patients with ischemic stroke who are at risk of poor adherence to home‐based functional exercises.

**Methods:**

We conducted a cross‐sectional study of 536 patients with ischemic stroke and limb dysfunction at a tertiary hospital in China. Latent profile analysis (LPA) was used to classify adherence patterns based on the Exercise Adherence Questionnaire. The least absolute shrinkage and selection operator (LASSO) regression was applied to select predictors from 35 candidate variables, and multivariable logistic regression was then used to build the prediction model. Model performance was internally validated using 1000 bootstrap resamples and assessed in terms of discrimination, calibration, and clinical utility. A web‐based nomogram was developed for clinical use.

**Results:**

The sample included 254 males (47.4%) and 282 females (52.6%); 61.0% were aged ≥ 60 years. LPA identified three adherence profiles: low (18.1%), moderate (42.2%), and high (39.7%). The optimal cutoff score for distinguishing good from poor adherence was 36.5 points. Five independent predictors were retained: marital status (never married: odds ratio [OR] = 0.03, 95% confidence interval [CI]: 0.01–0.11), monthly income > 5000 RMB (OR = 0.31, 95% CI: 0.14–0.67), spouse as primary caregiver (OR = 0.23, 95% CI: 0.10–0.53), knowledge level (OR = 0.92, 95% CI: 0.87–0.98), and exercise motivation (OR = 0.87, 95% CI: 0.81–0.93). Internal validation using 1000 bootstrap resamples showed good discrimination (apparent C‐statistic = 0.858; optimism‐corrected C‐statistic = 0.848), good calibration (optimism‐corrected calibration slope = 0.939; calibration intercept = 0.009), and a positive net benefit across threshold probabilities ranging from 0 to 0.80. After uniform shrinkage, model performance remained stable.

**Conclusions:**

We developed and internally validated a prediction model combining LPA with LASSO–logistic regression. The web‐based nomogram may facilitate early identification of patients at risk of poor adherence to home‐based functional exercises and support targeted interventions to improve rehabilitation outcomes.

## Graphical abstract

We developed and internally validated a web‐based nomogram to predict poor adherence to home‐based functional exercise after ischemic stroke. In 536 patients, latent profile analysis identified three adherence profiles, and a five‐predictor LASSO–logistic model demonstrated good discrimination, calibration, and clinical utility.

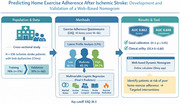

**Abbreviations**
aBIC: adjusted Bayesian information criterion
AIC: Akaike information criterion
AUC: area under the curve
BIC: Bayesian information criterion
BLRT: bootstrap likelihood ratio Test
BREQ‐3: Behavioral Regulation in Exercise Questionnaire‐3
DCA: decision curve analysis
EA: exercise adherence
EAQ: Exercise Adherence Questionnaire
GSES: General Self‐Efficacy Scale
IMB: information–motivation–behavioral skills
IS: ischemic stroke
LASSO: least absolute shrinkage and selection operator
LMRT: Lo–Mendell–Rubin adjusted likelihood ratio test
LPA: latent profile analysis
OR: odds ratio
PSSS: Perceived Social Support Scale
RAI: relative autonomy index
ROC: receiver operating characteristic
SKQ: Stroke Knowledge Questionnaire

## 1 Introduction

Stroke, caused by arterial occlusion or rupture, leads to cerebral ischemia or hemorrhage and subsequent neurological deficits. Because of its high incidence, disability, recurrence, and mortality, stroke remains a major global public health challenge (S. Wu et al. 2019). The burden is particularly substantial in China, where stroke contributes markedly to mortality, disability, and health‐care costs (Ma et al. 2021). Ischemic stroke (IS) is the most common subtype and is frequently followed by motor dysfunction, which often requires prolonged rehabilitation (Feske 2021; Walter 2022).

Although inpatient and outpatient rehabilitation can promote recovery, optimal long‐term outcomes usually depend on continued exercise after discharge. Home‐based functional exercise is therefore an important component of stroke rehabilitation because it can complement formal rehabilitation services and increase the overall dose of practice needed to support neuroplasticity, functional recovery, and quality of life (Ding et al. 2022; Gurková et al. 2023). However, the effectiveness of home‐based functional exercise depends largely on adherence. Poor adherence may delay recovery, increase complications and readmissions, and intensify caregiver burden, making early identification of individuals at risk of poor adherence clinically important (Mahmood et al. 2022; Mayo 2016; Y. Zhang et al. 2023).

Adherence to home‐based functional exercise after stroke is influenced by multiple factors, including sociodemographic characteristics, clinical conditions, and psychosocial resources (Gwynne‐Mayer et al. 2025). Previous studies have shown that adherence may be associated with age, education, financial status, environmental context, comorbidities, and discharge instruction, as well as modifiable psychosocial factors such as knowledge, motivation, self‐efficacy, and social support (B. Lin et al. 2023). Nevertheless, most existing studies have focused on describing adherence levels, trajectories, or associated factors rather than enabling early risk stratification for poor adherence (Arensman et al. 2023; Xing et al. 2025; Y. Zhang et al. 2023).

Current assessments mainly rely on self‐report scales and single time‐point questionnaire scores. Although these measures are useful for describing adherence, they are less suitable for rapid screening and risk prediction in routine clinical and follow‐up settings. Few studies have developed and validated practical prediction tools to support early identification of patients at risk of poor adherence to home‐based functional exercise after stroke. This gap limits the clinical translatability of existing evidence and the implementation of precision rehabilitation management.

To address this gap, we aimed to (1) use latent profile analysis (LPA) to identify adherence classes and define class‐assignment thresholds, (2) select key predictors using the least absolute shrinkage and selection operator (LASSO) regression, and (3) develop a multivariable logistic regression model to predict adherence to home‐based functional exercises. We further developed a web‐based tool to facilitate rapid risk identification and support risk‐stratified management in clinical and follow‐up settings.

### 1.1 Theoretical Framework

This study was informed by the information–motivation–behavioral skills (IMB) model, which posits that the initiation and maintenance of health behaviors depend on three components: accurate information, personal and social motivation, and the behavioral skills needed to enact and sustain the behavior (H. Xu and Wang 2023). In this context, information includes knowledge about stroke and rehabilitation (e.g., exercise goals, safe dosing, and expected benefits); motivation includes attitudes, outcome expectancies, and social support from family and the community; and behavioral skills encompass action planning, self‐monitoring, problem‐solving, and self‐efficacy under challenging conditions. Guided by the IMB model, we selected candidate variables and developed a LASSO–logistic regression prediction model.

## 2 Methods

### 2.1 Design and Setting

We conducted a cross‐sectional observational study using convenience sampling at the Second Affiliated Hospital of Harbin Medical University between May and July 2025. Participants included inpatients receiving rehabilitation and outpatients attending follow‐up visits. The methods and results are reported in accordance with the Strengthening the Reporting of Observational Studies in Epidemiology (STROBE) statement for cross‐sectional studies (Vandenbroucke et al. 2007). To enhance transparency and reproducibility, we developed a prespecified standard operating procedure and provided standardized training to all research staff.

### 2.2 Ethical Consideration

Before data collection, trained researchers explained the study aims, procedures, potential risks and benefits, confidentiality protections, and the voluntary nature of participation. Written informed consent was obtained from each participant or a legally authorized representative. The protocol was approved by the Ethics Committee of the Second Affiliated Hospital of Harbin Medical University (Approval No.: KY2025‐169) and was conducted in accordance with the Declaration of Helsinki.

### 2.3 Participants

Inclusion criteria: (1) age ≥ 18 years, (2) IS confirmed by cranial CT or MRI, (3) limb functional impairment with clinical stability after the acute treatment stage and ability to participate in home‐based functional exercise, and (4) ability to complete the questionnaire independently or with assistance. Exclusion criteria: (1) severe comorbid disease (e.g., advanced cardiac, pulmonary, or renal failure, or malignancy) that hindered participation, (2) marked cognitive impairment or psychiatric disorders that precluded valid assessment, and (3) severe communication or reading impairments.

### 2.4 Sample Size Estimation

Sample size followed the rule of at least 10 events per variable (van Smeden et al. 2019). With 35 candidate variables, a minimum of 350 events in the development data was considered necessary to support model robustness and generalizability. Based on available resources and the accessible population, we collected 536 valid questionnaires, which met this requirement.

### 2.5 Data Collection

Data were collected through face‐to‐face interviews using structured paper‐based questionnaires administered by trained interviewers. For participants with limited literacy or upper‐limb motor impairments, interviewers read items aloud and recorded responses according to a standardized protocol. All completed questionnaires were verified on site for completeness and consistency, and any missing data were clarified before the interview concluded. To ensure data accuracy, two independent researchers performed double data entry, followed by cross‐checking to identify and resolve discrepancies.

### 2.6 Measures

#### 2.6.1 General Information Questionnaire

We collected sociodemographic and clinical characteristics, including sex, age, education, marital status, pre‐illness employment, monthly income, medical payment type, living arrangement, household registration, family history of disease, primary caregiver, presence of chronic comorbidities, number of stroke events, receipt of discharge rehabilitation instruction, smoking history, and alcohol use.

#### 2.6.2 Exercise Adherence Questionnaire (EAQ)

The EAQ, developed by Lin et al. (2013), measures adherence to functional exercise during stroke rehabilitation. It includes 14 items across three domains: participation in exercise, monitoring of exercise outcomes, and seeking exercise guidance. Items are rated on a 4‐point Likert scale from 1 (*Not at all possible*) to 4 (*Completely possible*), yielding total scores from 14 to 56; higher scores indicate better adherence. Internal consistency in this study was good (Cronbach's *α* = 0.875).

#### 2.6.3 Stroke Knowledge Questionnaire (SKQ)

The SKQ, developed by Yao (2016), assesses stroke‐related knowledge across six domains: emergency response, warning signs, risk factors, healthy behaviors, rehabilitation, and medication safety. The 40 items are scored dichotomously (1 = correct; 0 = incorrect/don't know), producing a total score of 0–40; higher scores reflect greater knowledge. Internal consistency in this study was good (Cronbach's *α* = 0.862).

#### 2.6.4 Behavioral Regulation in Exercise Questionnaire‐3 (BREQ‐3)

The BREQ‐3 comprises 24 items covering six forms of motivation: intrinsic regulation, integrated regulation, identified regulation, introjected regulation, external regulation, and amotivation (four items each) (Markland and Tobin 2010). Items are rated on a 5‐point Likert scale from 0 (*Not true at all*) to 4 (*Very true*). Subscale scores are calculated by summing the four items for each regulation (range 0–16), and the Relative Autonomy Index (RAI) is calculated as: RAI = (3 × intrinsic) + (2 × integrated) + identified − introjected − (2 × external) − (3 × amotivation). Higher RAI values indicate more autonomous motivation. The Chinese version translated by Fan (2018) showed good psychometric properties in university students (Cronbach's *α* = 0.828). In this study, internal consistency was acceptable (Cronbach's *α* = 0.721).

#### 2.6.5 General Self‐Efficacy Scale (GSES)

The GSES evaluates confidence in managing daily demands and challenges (J. Zhang and Schwarzer 1995). It includes 10 items rated on a 4‐point Likert scale from 1 (*Not at all true*) to 4 (*Exactly true*), producing a total score of 10–40; higher scores indicate stronger self‐efficacy. Internal consistency in this study was good (Cronbach's *α* = 0.810).

#### 2.6.6 Perceived Social Support Scale (PSSS)

The PSSS measures perceived social support across three subscales: family (items 3, 4, 8, 11), friends (items 6, 7, 9, 12), and significant others/other support (items 1, 2, 5, 10) (Dahlem et al. 1991). Items are rated on a 7‐point Likert scale from 1 (*Very strongly disagree*) to 7 (*Very strongly agree*), yielding total scores of 12–84; higher scores denote greater perceived support. In this study, internal consistency was good (Cronbach's *α* = 0.888).

### 2.7 Statistical Analysis

Data entry, descriptive statistics, and group comparisons were performed using SPSS Statistics 27.0 (IBM Corp., Armonk, NY, USA). LPA was conducted using Mplus 8.3 (Muthén & Muthén, Los Angeles, CA, USA). Model development, internal validation, and performance evaluation were conducted in R 4.3.1 (R Foundation for Statistical Computing, Vienna, Austria). Because data were collected using self‐report questionnaires, common method variance was assessed using Harman's single‐factor test (Podsakoff et al. 2003). Continuous variables were examined for approximate normality using skewness and kurtosis and are presented as mean ± standard deviation (SD), while categorical variables are presented as frequencies and percentages (Rockwood 2021).

LPA was used to identify latent adherence subgroups based on responses to the 14 EAQ items. One‐ to five‐class models were fitted, and the optimal model was selected according to statistical fit and clinical interpretability (X. Wang et al. 2022). Model selection considered Akaike information criterion (AIC), Bayesian information criterion (BIC), adjusted BIC (aBIC), entropy, and the Lo–Mendell–Rubin adjusted likelihood ratio test (LMRT) and bootstrap likelihood ratio test (BLRT). The identified adherence classes were then dichotomized into good adherence and poor adherence groups, and the optimal EAQ cutoff score was determined using receiver operating characteristic (ROC) analysis and the maximum Youden index.

For model development, the full dataset was used after predictor selection and confirmation. Baseline characteristics were compared using Pearson's chi‐square test or Fisher's exact test, as appropriate. LASSO regression was used to identify candidate predictors of poor adherence. Variables retained by LASSO were further examined using univariable logistic regression, and eligible variables were entered into a multivariable logistic regression model to estimate odds ratios (ORs) and 95% confidence intervals (CIs). Internal validation was performed using bootstrap resampling with 1000 repetitions to reduce the risk of overfitting and to obtain more robust estimates of model performance. Model optimism was quantified, and optimism‐corrected estimates of discrimination and calibration were calculated. Discrimination was assessed using the C‐statistic. Calibration was assessed using the calibration slope, calibration intercept, and a bootstrap‐based calibration plot. To account for potential overfitting, uniform shrinkage was performed using the optimism‐corrected calibration slope as the shrinkage factor, followed by reestimation of the model intercept. Clinical utility was evaluated using decision curve analysis (DCA). A two‐sided *p* value < 0.05 was considered statistically significant. As a supplementary sensitivity analysis, a random 70/30 split‐sample evaluation was also performed to examine the robustness of the model findings.

### 2.8 Web‐Based Dynamic Nomogram

Based on the final multivariable logistic regression model, an interactive web‐based dynamic nomogram was developed using the DynNom package within the Shiny framework and deployed via shinyapps.io to support rapid risk estimation in clinical and follow‐up settings.

## 3 Results

### 3.1 Common Method Deviation Test

Harman's single‐factor analysis yielded four factors with eigenvalues > 1. The first factor explained 38.614% of the total variance, which was below the conventional 40% threshold, suggesting that common method bias was unlikely to meaningfully affect the results.

### 3.2 General Demographic Characteristics

A total of 536 patients with limb dysfunction after IS were included: 254 men (47.4%) and 282 women (52.6%). Most participants were married and enrolled in urban medical insurance, and spouses or adult children were most frequently identified as the primary caregivers. Detailed characteristics are provided in Table S1.

### 3.3 Descriptive Statistics of Exercise Adherence (EA) and Related Psychosocial Measures

The mean total EAQ score was 34.64 ± 7.94. Among the three domains, mean scores from highest to lowest were exercise participation (20.11 ± 4.55), monitoring of exercise outcomes (7.55 ± 2.24), and seeking exercise guidance (6.98 ± 2.57). The mean total SKQ score was 28.40 ± 7.10, with domain scores of emergency response (5.71 ± 1.78), warning signs (2.88 ± 1.16), risk factors (8.01 ± 2.76), healthy behaviors (2.89 ± 1.08), rehabilitation knowledge (6.74 ± 2.33), and medication safety (2.18 ± 0.96). The mean BREQ‐3 subscale sum scores were amotivation (8.49 ± 3.66), external regulation (8.59 ± 3.78), introjected regulation (8.53 ± 3.59), identified regulation (8.32 ± 3.81), integrated regulation (8.92 ± 3.74), and intrinsic regulation (8.87 ± 3.88); based on these six subscales, the RAI was −0.41 ± 5.12. The mean total GSES score was 27.49 ± 6.92. The mean total PSSS score was 53.83 ± 13.63, with subscale scores of family support (17.79 ± 5.95), friend support (18.14 ± 5.91), and significant others/other support (17.90 ± 6.00).

### 3.4 LPA of EA in Patients With Limb Dysfunction due to IS

LPA was conducted using responses to the 14 EAQ items assessing adherence to home‐based functional exercises, and one‐ to five‐class models were fitted (Table S2). The information criteria (AIC, BIC, aBIC) decreased as the number of classes increased; however, the three‐class model provided the best balance between statistical fit and clinical interpretability. Although the four‐ and five‐class models further reduced the information criteria, they yielded small classes with limited clinical interpretability. The LMRT and BLRT supported solutions up to three classes (*p* < 0.001). The LMRT was marginal for the four‐class model and nonsignificant for the five‐class model. Average posterior probabilities were high—97.4% (Profile 1), 93.7% (Profile 2), and 94.7% (Profile 3)—exceeding the 90% benchmark and indicating stable classification (Table S3).

**FIGURE 1:**
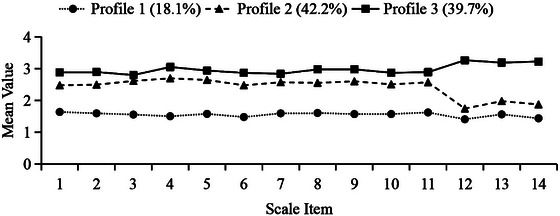
Latent profile analysis of adherence to home‐based functional exercise in patients with ischemic stroke–related limb dysfunction. Three latent profiles were identified from the 14 items of the Exercise Adherence Questionnaire (EAQ). The *x*‐axis shows the scale items, and the *y*‐axis shows the mean item scores. Profile 1 represented low adherence (18.1%), Profile 2 moderate adherence (42.2%), and Profile 3 high adherence (39.7%).

The three profiles showed distinct item‐response patterns (Figure 1). Profile 1 (“low adherence,” *n* = 97, 18.1%) showed uniformly low mean scores across the 14 items, and Profile 3 (“high adherence,” *n* = 213, 39.7%) showed consistently high mean scores. Profile 2 (“moderate adherence,” *n* = 226, 42.2%) had intermediate overall levels; however, its item means were not fully parallel to those of Profiles 1 and 3, with a notable decrease at Item 12.

### 3.5 Identification of Predictors of EA

Based on the LPA results, the “high‐adherence” class (*n* = 213) was labeled “good adherence,” whereas the “low” and “moderate” classes (*n* = 323) were combined as “poor adherence.” Using this binary outcome, we generated an ROC curve for the EAQ and evaluated candidate cutoff scores (Table S4). The optimal cutoff score was 36.5 (Youden index = 0.789). Using this cutoff, the estimated prevalence of poor adherence in this sample was 51.31%.

Table 1 presents the results of the supplementary 70/30 split‐sample comparison by adherence group. In this supplementary analysis, several sociodemographic and psychosocial variables differed between adherence groups, including marital status, living arrangement, primary caregiver, participation in discharge rehabilitation instruction, knowledge, exercise motivation, self‐efficacy, and social support.

**TABLE 1:** Supplementary comparison of general characteristics of participants in the 70/30 split‐sample training and validation sets.

Feature	Training set (*N* = 376)		*p*	Internal validation set (*N* = 160)		*p*
	Non‑good adherence (*N* = 229)	Good adherence (*N* = 147)		Non‑good adherence (*N* = 94)	Good adherence (*N* = 66)	
Gender			0.544			0.503
Male	102 (44.5%)	71 (48.3%)		45 (47.9%)	36 (54.5%)	
Female	127 (55.5%)	76 (51.7%)		49 (52.1%)	30 (45.5%)	
Age			0.681			0.502
< 50	44 (19.2%)	33 (22.4%)		15 (16.0%)	14 (21.2%)	
50–59	39 (17.0%)	30 (20.4%)		19 (20.2%)	15 (22.7%)	
60–69	40 (17.5%)	26 (17.7%)		18 (19.1%)	12 (18.2%)	
70–79	58 (25.3%)	29 (19.7%)		14 (14.9%)	13 (19.7%)	
≥ 80	48 (21.0%)	29 (19.7%)		28 (29.8%)	12 (18.2%)	
Education level			0.884			0.953
Primary school or below	51 (22.3%)	28 (19.0%)		28 (29.8%)	20 (30.3%)	
Junior high school	47 (20.5%)	33 (22.4%)		18 (19.1%)	12 (18.2%)	
Senior high school or technical secondary school	61 (26.6%)	39 (26.5%)		21 (22.3%)	17 (25.8%)	
College degree or above	70 (30.6%)	47 (32.0%)		27 (28.7%)	17 (25.8%)	
Marital status			**< 0.001**			**0.003**
Never married	10 ( 4.4%)	41 (27.9%)		9 ( 9.6%)	18 (27.3%)	
Married	110 (48.0%)	61 (41.5%)		40 (42.6%)	30 (45.5%)	
Divorced/Widowed	109 (47.6%)	45 (30.6%)		45 (47.9%)	18 (27.3%)	
Pre‐illness employment status			0.846			0.521
Unemployed	71 (31.0%)	46 (31.3%)		29 (30.9%)	15 (22.7%)	
Retired	84 (36.7%)	50 (34.0%)		34 (36.2%)	26 (39.4%)	
Employed	74 (32.3%)	51 (34.7%)		31 (33.0%)	25 (37.9%)	
Monthly income			0.097			0.223
< 2000	69 (30.1%)	35 (23.8%)		30 (31.9%)	13 (19.7%)	
2000–5000	88 (38.4%)	50 (34.0%)		34 (36.2%)	27 (40.9%)	
> 5000	72 (31.4%)	62 (42.2%)		30 (31.9%)	26 (39.4%)	
Type of medical payment			0.285			0.605
Provincial health insurance	60 (26.2%)	32 (21.8%)		19 (20.2%)	15 (22.7%)	
Municipal health insurance	57 (24.9%)	47 (32.0%)		28 (29.8%)	23 (34.8%)	
New Rural Cooperative Medical Scheme	61 (26.6%)	31 (21.1%)		24 (25.5%)	11 (16.7%)	
Out‐of‐pocket payment	51 (22.3%)	37 (25.2%)		23 (24.5%)	17 (25.8%)	
Living arrangement			**0.017**			0.210
Living alone	61 (26.6%)	19 (12.9%)		20 (21.3%)	9 (13.6%)	
Living with family	55 (24.0%)	40 (27.2%)		22 (23.4%)	25 (37.9%)	
Living with a caregiver/domestic helper	56 (24.5%)	43 (29.3%)		22 (23.4%)	15 (22.7%)	
Living in a nursing home/other institution	57 (24.9%)	45 (30.6%)		30 (31.9%)	17 (25.8%)	
Household registration type			0.934			0.098
Rural household registration	113 (49.3%)	74 (50.3%)		39 (41.5%)	37 (56.1%)	
Urban household registration	116 (50.7%)	73 (49.7%)		55 (58.5%)	29 (43.9%)	
Family medical history			1.000			0.978
Yes	118 (51.5%)	71 (48.3%)		47 (50.0%)	34 (51.5%)	
No	111 (48.5%)	76 (51.7%)		47 (50.0%)	32 (48.5%)	
Primary caregiver			**0.016**			0.184
Spouse	51 (22.3%)	45 (30.6%)		27 (28.7%)	26 (39.4%)	
Children	66 (28.8%)	47 (32.0%)		18 (19.1%)	17 (25.8%)	
Caregiver/domestic helper	48 (21.0%)	34 (23.1%)		23 (24.5%)	12 (18.2%)	
Others (e.g., parents, friends)	64 (27.9%)	21 (14.3%)		26 (27.7%)	11 (16.7%)	
Presence of comorbid chronic diseases			0.070			**0.025**
Yes	126 (55.0%)	66 (44.9%)		58 (61.7%)	28 (42.4%)	
No	103 (45.0%)	81 (55.1%)		36 (38.3%)	38 (57.6%)	
Number of strokes			0.068			**< 0.001**
1	63 (27.5%)	48 (32.7%)		14 (14.9%)	30 (45.5%)	
2	56 (24.5%)	42 (28.6%)		28 (29.8%)	12 (18.2%)	
3	57 (24.9%)	39 (26.5%)		30 (31.9%)	13 (19.7%)	
≥ 4	53 (23.1%)	18 (12.2%)		22 (23.4%)	11 (16.7%)	
Participation in discharge rehabilitation instruction			**0.048**			**0.038**
Participated	101 (44.1%)	81 (55.1%)		43 (45.7%)	42 (63.6%)	
Not participated	128 (55.9%)	66 (44.9%)		51 (54.3%)	24 (36.4%)	
Smoke			0.094			0.285
Yes	129 (56.3%)	69 (46.9%)		42 (44.7%)	36 (54.5%)	
No	100 (43.7%)	78 (53.1%)		52 (55.3%)	30 (45.5%)	
Drink			1.000			0.060
Yes	100 (43.7%)	64 (43.5%)		51 (54.3%)	25 (37.9%)	
No	129 (56.3%)	83 (56.5%)		43 (45.7%)	41 (62.1%)	
Knowledge level [M ± SD]	26.03 ± 7.41	31.99 ± 4.39	**< 0.001**	25.78 ± 7.70	32.36 ± 4.43	**< 0.001**
Exercise motivation [M ± SD]	−2.15 ± 3.88	2.28 ± 5.39	**< 0.001**	−2.31 ± 3.69	2.41 ± 6.11	**< 0.001**
Self‐efficacy [M ± SD]	25.15 ± 6.59	31.10 ± 5.81	**< 0.001**	25.07 ± 6.84	31.05 ± 5.40	**< 0.001**
Social support [M ± SD]	50.07 ± 12.93	60.02 ± 11.79	**< 0.001**	48.90 ± 14.42	60.09 ± 11.02	**< 0.001**

*Note*: Data are presented as *n* (%) unless otherwise indicated. Continuous variables are presented as M ± SD. Bold values indicate statistical significance (*p* < 0.05). *p* values were derived from Pearson's chi‐square test or Fisher's exact test, as appropriate.

Abbreviations: IS, ischemic stroke; M, mean; SD, standard deviation.

With poor adherence (vs. good adherence) as the binary outcome, we applied LASSO regression to identify key predictors. Ten variables were retained: never‑married status; monthly income > 5000; living alone; primary caregiver (spouse, children, caregiver/domestic helper); and four continuous measures—knowledge, exercise motivation, self‑efficacy, and social support (Figure S1). These variables were subsequently entered into multivariable modeling.

### 3.6 Development of the EA Predictive Model

Predictors selected by LASSO were entered into univariable and multivariable logistic regression models to confirm associations and develop the prediction model. Five variables remained statistically significant in the final multivariable model (all *p* < 0.05): marital status, monthly income, primary caregiver, knowledge level, and exercise motivation. Full results are presented in Table 2. Using the regression coefficients from the final model, we developed an interactive, web‐based dynamic nomogram to support clinical applications (https://wenboli.shinyapps.io/dynnomapp/).

**TABLE 2:** Final multivariable logistic regression model for predicting poor exercise adherence.

Predictors	*N*	No adherence (*n*)	OR	95% CI	*p*
Marital status					
Married	171	110	—	—	—
Never married	51	10	0.03	0.01, 0.11	**< 0.001**
Divorced/widowed	154	109	1.04	0.58, 1.89	0.887
Monthly income					
<2000	104	69	—	—	—
2000–5000	138	88	0.45	0.20, 0.99	**0.047**
>5000	134	72	0.31	0.14, 0.67	**0.003**
Living arrangement					
Living with family	95	55	—	—	—
Living alone	80	61	1.97	0.83, 4.71	0.126
Living with a caregiver/domestic helper	99	56	0.67	0.32, 1.43	0.304
Living in a nursing home/other institution	102	57	0.64	0.30, 1.36	0.246
Primary caregiver					
Others (e.g., parents, friends)	85	64	—	—	—
Spouse	96	51	0.23	0.10, 0.53	**< 0.001**
Children	113	66	0.29	0.13, 0.68	**0.004**
Caregiver/domestic helper	82	48	2.08	0.72, 6.02	0.177
Knowledge level [M(IQR)]	—	—	0.92	0.87, 0.98	**0.009**
Exercise motivation [M(IQR)]	—	—	0.87	0.81, 0.93	**< 0.001**
Self‐efficacy [M(IQR)]	—	—	0.96	0.91, 1.02	0.206
Social support [M(IQR)]	—	—	0.98	0.95, 1.01	0.158

*Note*: Bold values indicate statistical significance (*p* < 0.05).

Abbreviations: CI, confidence interval; IQR, interquartile range; M, mean; *n*, number of participants with poor adherence; *N*, total number of participants; OR, odds ratio; Ref, reference category.

### 3.7 Validation of the EA Predictive Model

**FIGURE 2:**
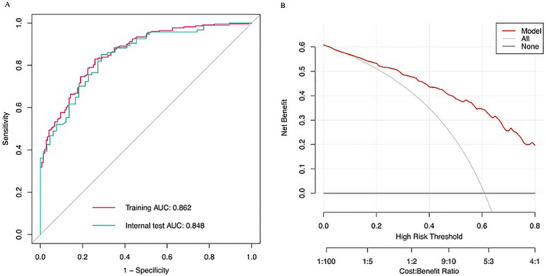
Supplementary performance of the predictive model in the random 70/30 split‐sample analysis. (A) ROC curves for the model in the split‐sample training and validation cohorts, with AUCs of 0.862 and 0.848, respectively. (B) Decision curve analysis showing the net benefit of the model across threshold probabilities.

Model performance was evaluated in terms of discrimination, calibration, and clinical utility. Internal validation was performed using 1000 bootstrap resamples. The apparent C‐statistic of the model was 0.858, and the optimism‐corrected C‐statistic was 0.848, indicating good discrimination with limited optimism. The optimism‐corrected calibration slope was 0.939 and the calibration intercept was 0.009, suggesting only mild overfitting and good overall agreement between predicted and observed risks. To further reduce overfitting, uniform shrinkage was applied using the optimism‐corrected calibration slope as the shrinkage factor (0.939), followed by reestimation of the intercept. After shrinkage, model performance remained stable (area under the curve [AUC] = 0.858; Brier score = 0.1498). The bootstrap‐based calibration plot demonstrated good agreement between predicted and observed probabilities. For clinical utility, DCA showed a positive net benefit across threshold probabilities of 0–0.80, consistently outperforming the reference strategies, supporting potential clinical usefulness within this range (Figure 2B).

## 4 Discussion

People living with the consequences of stroke may experience delayed functional recovery, depressive symptoms, and increased caregiver burden, which are often associated with poor adherence during rehabilitation (Keser et al. 2023; J. Wang et al. 2024). Early identification of individuals at high risk of poor adherence is critical for delivering targeted and timely interventions. To our knowledge, this study is the first to develop a prediction model for adherence to home‐based functional exercises among patients with IS. By integrating LPA, LASSO regression, and multivariable logistic regression, we identified key predictors and developed an interpretable, web‐based prediction tool. Our findings may also be understood in the context of emerging nomogram‐based prediction studies in stroke populations. For example, Y. Wu et al. (2025) developed a dynamic nomogram for predicting fall risk in stroke patients, demonstrating that individualized and web‐based prediction tools may support risk stratification and clinical decision‐making in stroke rehabilitation. Compared with that study, the present study extends the application of dynamic nomogram methodology from fall‐risk prediction to adherence to home‐based functional exercises, an outcome that is highly relevant to long‐term rehabilitation engagement and functional recovery. This comparison further supports the feasibility and clinical plausibility of using nomogram‐based tools in stroke care. In addition, methodological guidance on multivariable prediction model development has emphasized the importance of adequate sample size planning, transparent model construction, and appropriate validation to enhance model stability and clinical applicability (Riley et al. 2019). In this context, our model incorporated data‐driven predictor selection, bootstrap‐based internal validation, and evaluation of discrimination, calibration, and clinical utility, thereby providing stronger methodological support for its use as an early screening tool in rehabilitation practice. The bootstrap analysis further showed only limited optimism, with an optimism‐corrected calibration slope of 0.939 and calibration intercept of 0.009, indicating mild overfitting and good overall agreement between predicted and observed risks. The model demonstrated favorable calibration and clinical utility, suggesting potential value for risk stratification and individualized rehabilitation management.

### 4.1 Potential Profile Characteristics of Home‐Based EA Types in Patients With Limb Dysfunction due to IS

LPA identified three distinct adherence profiles—low, moderate, and high—highlighting heterogeneity in adherence to home‐based functional exercises among people with limb dysfunction after IS. This classification is consistent with previous studies reporting low–moderate–high typologies of health behaviors in high‐risk populations (Guo et al. 2020). In our sample, the moderate‐ and high‐adherence profiles accounted for 81.9% of participants, suggesting that most maintained at least a moderate level of engagement. However, 18.1% were classified as low adherence, a group at heightened risk of poor recovery and long‐term disability (Billinger et al. 2014). Prior work similarly indicates that low adherence is associated with functional decline, depressive symptoms, and greater caregiver burden (Arensman et al. 2024; Schuch and Stubbs 2019). These findings underscore the importance of early identification and targeted interventions for individuals with low adherence, such as structured rehabilitation instruction and ongoing feedback to enhance engagement (Milton‐Cole et al. 2024; Soong et al. 2025).

Based on evidence from prior research, patients classified as having “low adherence” have been reported to be less likely to seek professional support, which may reflect lower health awareness, self‐efficacy, or social support (Xie et al. 2020). Patients in the “moderate adherence” profile showed more consistent exercise behaviors but appeared less inclined to actively seek rehabilitation information (Milani et al. 2024; Salvi et al. 2018; Yen et al. 2023). By contrast, those in the “high adherence” group demonstrated stronger self‐management and resource utilization, patterns commonly associated with higher knowledge levels, stronger self‐efficacy, and greater perceived social support (Shi et al. 2025; Teixeira et al. 2012). From a nursing perspective, reinforcing effective behaviors and promoting peer‐to‐peer experience sharing may help foster behavioral improvement among individuals with low adherence (Patil et al. 2018).

### 4.2 Predictors of EA

#### 4.2.1 Marital Status

Married participants were more likely to achieve good adherence to home‐based functional exercises, underscoring the role of spousal support during early rehabilitation. Prior studies indicate that marital relationships can strengthen confidence and persistence by providing emotional encouragement and practical assistance (Z. Wang et al. 2025). Spousal and broader family support may also promote self‐management through reassurance, supervision, and instrumental aid. In addition, dyadic interventions involving both patients and caregivers have been shown to improve adherence and quality of life in other clinical populations (Shen et al. 2025; Son et al. 2025). Our findings extend this evidence to IS and suggest that assessment of family support structures may be important when evaluating adherence risk. For individuals who are single, divorced, or widowed, alternative supports (e.g., volunteer companionship or community‐based rehabilitation programs) may help compensate for reduced spousal support.

#### 4.2.2 Monthly Income

Higher monthly income was associated with better adherence. Financial resources can affect access to rehabilitation services and the ability to sustain training over time. A multicenter study reported that financial strain was associated with discontinuation of post‐stroke rehabilitation, with lower income groups being particularly prone to dropout (L. Xu et al. 2024). Our findings are consistent with this evidence, suggesting that affordability is an important determinant of adherence in home‐based functional exercise programs. In practice, individuals with limited resources may benefit from low‐cost, feasible exercise prescriptions (e.g., equipment‐free routines or community‐based options). Expanding insurance coverage or providing targeted subsidies may also reduce income‐related disparities.

#### 4.2.3 Primary Caregiver

Primary caregiver status was assessed using the General Information Questionnaire and categorized as spouse, adult children, caregiver/domestic helper, or other. Participants whose primary caregiver was a spouse or adult child showed better adherence than those primarily supported by nonfamily caregivers, highlighting the potential importance of long‐term companionship and emotional bonds. Prior work suggests that active caregiver involvement is associated with better engagement and outcomes; for example, committed caregivers and caregiver training have been linked to higher odds of community discharge after rehabilitation (Bosch et al. 2023). Our findings extend this evidence by suggesting that caregiver type may matter: compared with family caregivers, hired caregivers may be associated with more variable adherence, possibly due to weaker shared goals or less emotional connection. Follow‐up management should therefore consider caregiver involvement and provide tailored guidance for different caregiver roles.

#### 4.2.4 Knowledge Level

In this study, stroke‐related knowledge was measured using the total SKQ score. Higher SKQ scores predicted better adherence to home‐based functional exercises. Low health literacy is consistently linked to poor self‐management and greater early post‐discharge utilization—for example, a 46% higher 30‐day reutilization rate among heart failure patients with inadequate health literacy (IRR = 1.46) (Mitchell et al. 2012). Among people affected by stroke, lower health literacy has been associated with poorer quality of life, with resilience and social support acting as mediators (Chen et al. 2024). In stroke rehabilitation, understanding the disease and training principles may help individuals appraise risks, set realistic goals, and avoid discontinuation driven by misconceptions. However, knowledge alone may be insufficient; without motivation or adequate support, information may not translate into sustained behavior. Interventions may therefore be more effective when education is combined with motivational enhancement and support‐system strengthening rather than relying on information provision alone.

#### 4.2.5 Exercise Motivation (BREQ‑3 RAI)

In this study, exercise motivation was operationalized using the RAI derived from the BREQ‐3, with higher RAI values indicating more autonomous motivation. Exercise motivation (BREQ‐3 RAI) was among the strongest predictors of adherence, suggesting that individuals with more autonomous motivation were more likely to maintain consistent adherence to home‐based functional exercises, consistent with self‐determination theory (Gangwani et al. 2022). Enhancing intrinsic motivation through specific goals and timely feedback may meaningfully improve adherence. For example, a survey of rehabilitation professionals reported widespread use of goal‐oriented practice (over 75%) to support motivation and exercise performance after stroke (Oyake et al. 2020). From a theoretical perspective, supporting autonomy and competence—through achievable goals and appropriately calibrated task difficulty—may facilitate internalization of motivation and promote sustained engagement (Fernandes et al. 2024). Our findings reinforce this perspective and highlight the importance of early assessment of motivation. For individuals with low autonomous motivation, approaches such as motivational interviewing, staged goal setting, and peer modeling may help transform external prompts into more durable, self‐determined motivation.

Overall, this study identified five independent predictors of adherence to home‐based functional exercises: marital status, monthly income, primary caregiver, knowledge level, and exercise motivation. In addition to these core variables, univariable analyses and LASSO suggested associations with education level, living arrangement, self‐efficacy, and perceived social support, which is consistent with prior literature on health literacy, environmental context, psychological resources, and social support. However, these factors were not retained in the final multivariable model, which may reflect partial mediation through knowledge or motivation, or potential interactions with marital status and caregiver type. Rather than diminishing their importance, this pattern may underscore the fact that adherence is influenced by multiple, potentially interrelated psychosocial factors.

### 4.3 Practical Implications

The proposed model may enable early identification of individuals at high risk of poor adherence, providing an evidence base for stratified rehabilitation management. Risk‐based stratification can help prioritize limited rehabilitation resources and follow‐up services for those with the greatest need, thereby improving the precision and efficiency of care. This approach may be particularly valuable in resource‐constrained settings, where optimized allocation can ensure that high‐need individuals receive appropriate support and, ultimately, improve rehabilitation quality and outcomes. Taken together with previous nomogram‐based work in stroke rehabilitation, these findings suggest that dynamic and clinically accessible prediction tools may have broader value in supporting individualized follow‐up and targeted intervention planning.

### 4.4 Strengths and Limitations

This study innovatively combined LPA with predictive modeling to characterize adherence patterns to home‐based functional exercises among people after stroke and to develop a risk prediction tool. By integrating LASSO regression, multivariable logistic regression, and a web‐based calculator, we developed a model with good calibration and clinical feasibility, providing a practical framework for adherence assessment in rehabilitation practice. Nevertheless, several limitations should be acknowledged. First, the cross‐sectional design precludes causal inference and does not permit assessment of longitudinal changes in adherence to home‐based functional exercises throughout the rehabilitation process. Given that adherence is a dynamic behavior that may vary over time, future longitudinal studies are warranted to examine temporal changes and their determinants across different rehabilitation phases. Second, convenience sampling from a single region may limit representativeness and generalizability; therefore, future studies should validate the model in more diverse populations and health‐care settings. Third, the current web‐based tool contains interface elements that are not fully in English, which may create a language barrier for some users and limit broader dissemination. Finally, although multiple psychosocial variables were included, other potentially important factors may not have been captured, which could have introduced residual confounding. These factors include cultural context, long‐term caregiving resources, and key clinical characteristics such as stroke severity, time since stroke onset, rehabilitation stage, prior rehabilitation exposure, and inpatient/outpatient care context. Although all included participants were clinically stable after the acute treatment stage and were able to engage in home‐based functional exercise, we did not further stratify them into subacute and chronic recovery stages, which may have influenced adherence patterns. Such factors may influence adherence to home‐based functional exercises and may also affect the identification and interpretation of latent adherence profiles. Future research should incorporate these variables, adopt longitudinal designs, and conduct stage‐stratified validation and extension of the model across different phases of rehabilitation.

## 5 Conclusion

By integrating LPA with LASSO‐assisted logistic regression, this study developed and internally validated a prediction model for adherence to home‐based functional exercises among people after stroke. The results revealed substantial heterogeneity across adherence subgroups and identified key predictors, including marital status, monthly income, primary caregiver, stroke‐related knowledge, and exercise motivation. The resulting web‐based dynamic nomogram may serve as a practical tool for risk stratification, supporting early identification of individuals at risk of poor adherence and informing tailored intervention strategies. Such approaches may improve adherence to rehabilitation, thereby promoting functional recovery and quality of life after stroke.

## Author Contributions

**Wenbo Li**: conceptualization, writing – original draft. **Qiujie Li**: data curation, writing – review and editing.

## Funding

Heilongjiang Provincial Natural Science Foundation of China: BS2025G001.

## Ethics Statement

This study was performed in line with the principles of the Declaration of Helsinki. Approval was granted by the Ethics Committee of the Second Affiliated Hospital of Harbin Medical University (Approval No.: KY2025‐169). All procedures performed in studies involving human participants were in accordance with the ethical standards of the institutional and/or national research committee and with the 1964 Helsinki declaration and its later amendments or comparable ethical standards.

## Consent

Informed consent was obtained from all individual participants included in the study. Written informed consent was obtained from all subjects before the study. Participants were fully informed about the purpose, procedures, potential risks and benefits of the study, and their right to withdraw from the study at any time without penalty.

## Conflicts of Interest

The authors declare no conflicts of interest.

## Supporting information




**Supporting Information**: brb371482‐sup‐0001‐SuppMat.docx

## Data Availability

The data that support the findings of this study are available from the corresponding author upon reasonable request.
