# Supplementary material for: Predicting Home Exercise Adherence after Ischemic Stroke: Development and Validation of a Web‐Based Nomogram
Source: Brain Behav. 2026 May 11;16(5):e71482. doi: 10.1002/brb3.71482 (PMC13159546; doi:10.1002/brb3.71482)
Supplement: Supplementary file 1 — Supporting Information: brb371482‐sup‐0001‐SuppMat.docx [file BRB3-16-e71482-s001.docx]

**Table S1 Participant characteristics**

| Characteristics | N (%) |
| --- | --- |
| **Gender** |  |
| Male | 254(47.4) |
| Female | 282(52.6) |
| **Age** |  |
| <50 | 106(19.8) |
| 50-59 | 103(19.2) |
| 60-69 | 96(17.9) |
| 70-79 | 114(21.3) |
| ≥80 | 117(21.8) |
| **Education level** |  |
| Primary school or below | 127(23.7) |
| Junior high school | 110(20.5) |
| Senior high school or technical secondary school | 138(25.7) |
| College degree or above | 161(30.0) |
| **Marital status** |  |
| Never married | 78(14.6) |
| Married | 241(45.0) |
| Divorced/Widowed | 217(40.5) |
| **Pre-illness employment status** |  |
| Unemployed | 161(30.0) |
| Retired | 194(36.2) |
| Employed | 181(33.8) |
| **Monthly income** |  |
| <2,000 | 147(27.4) |
| 2,000-5,000 | 199(37.1) |
| >5,000 | 190(35.4) |
| **Type of medical payment** |  |
| Provincial health insurance | 126(23.5) |
| Municipal health insurance | 155(28.9) |
| New Rural Cooperative Medical Scheme | 127(23.7) |
| Out-of-pocket payment | 128(23.9) |
| **Living arrangement** |  |
| Living alone | 109(20.3) |
| Living with family | 142(26.5) |
| Living with a caregiver/domestic helper | 136(25.4) |
| Living in a nursing home/other institution | 149(27.8) |
| **Household registration type** |  |
| Rural household registration | 263(49.1) |
| Urban household registration | 273(50.9) |
| **Family medical history** |  |
| Yes | 275(51.3) |
| No | 261(48.7) |
| **Primary caregiver** |  |
| Spouse | 149(27.8) |
| Children | 148(27.6) |
| Caregiver/domestic helper | 117(21.8) |
| Others (e.g., parents, friends) | 122(22.8) |
| **Presence of comorbid chronic diseases** |  |
| Yes | 278(51.9) |
| No | 258(48.1) |
| **Number of strokes** |  |
| 1 | 155(28.9) |
| 2 | 138(25.7) |
| 3 | 139(25.9) |
| ≥4 | 104(19.4) |
| **Participation in discharge rehabilitation instruction** |  |
| Participated | 267(49.8) |
| Not participated | 269(50.2) |
| **Smoke** |  |
| Yes | 276(51.5) |
| No | 260(48.5) |
| **Drink** |  |
| Yes | 240(44.8) |
| No | 296(55.2) |
| **Knowledge level [M ± SD]** | 28.40±7.10 |
| **Exercise motivation [M ± SD]** | -0.41±5.12 |
| **Self-efficacy [M ± SD]** | 27.49±6.92 |
| **Social support [M ± SD]** | 53.83±13.63 |

Table S2 Model fit indices for latent profiles of home-based functional exercise adherence among patients with limb dysfunction after ischemic stroke.

| Profile | k | Likelihood | AIC | BIC | aBIC | Entropy | LMRT(P) | BLRT(P) | Proportion |
| --- | --- | --- | --- | --- | --- | --- | --- | --- | --- |
| 1 | 28 | -9968.886 | 19993.772 | 20113.727 | 20024.846 | — | — | — | — |
| 2 | 43 | -9075.241 | 18236.482 | 18420.7 | 18284.203 | 0.909 | ＜0.001 | ＜0.001 | 0.37/0.63 |
| **3** | **58** | **-8772.51** | **17661.02** | **17909.5** | **17725.388** | **0.887** | **＜0.001** | **＜0.001** | **0.18/0.42/0.40** |
| 4 | 73 | -8636.025 | 17418.05 | 17730.792 | 17499.066 | 0.887 | 0.0055 | ＜0.001 | 0.30/0.16/0.14/0.39 |
| 5 | 88 | -8557.604 | 17291.208 | 17668.212 | 17388.871 | 0.882 | 0.068 | ＜0.001 | 0.16/0.21/0.29/0.22/0.12 |

Note: k = free parameters; AIC = Akaike information criterion; BIC = Bayesian information criterion; aBIC = adjusted BIC; LMRT = Lo-Mendell-Rub test; BLRT = Bootstrap Likelihood ratio test.

Table S3 Average posterior probabilities of class membership for each latent profile.

| Class | Profile 1 | Profile 2 | Profile 3 |
| --- | --- | --- | --- |
| Profile 1 | 0.974 | 0.026 | 0.000 |
| Profile 2 | 0.024 | 0.937 | 0.039 |
| Profile 3 | 0.000 | 0.053 | 0.947 |

**Table S4 Diagnostic metrics for candidate cutoff scores of the Exercise Adherence Questionnaire based on ROC analysis**

| **Cutoff Score** | **Sensitivity** | **Specificity** | **Youden Index** |
| --- | --- | --- | --- |
| 34.5 | 0.715 | 0.991 | 0.706 |
| 35.5 | 0.765 | 0.986 | 0.751 |
| **36.5** | **0.827** | **0.962** | **0.789** |
| 37.5 | 0.861 | 0.925 | 0.786 |
| 38.5 | 0.913 | 0.836 | 0.749 |
| 39.5 | 0.954 | 0.742 | 0.696 |
| 40.5 | 0.966 | 0.638 | 0.604 |

**Figure S1 LASSO regression feature selection results**

Note: Lasso Regression Path for Coefficient Shrinkage Across Varying Log Lambda Values. The optimal parameter [λ] selection in the LASSO model employed 10-fold cross-validation using a minimum criteria approach. The optimal values of λ are represented by dotted vertical lines.
